# Cutting-Edge Analysis of Extracellular Microparticles using ImageStream^X^ Imaging Flow Cytometry

**DOI:** 10.1038/srep05237

**Published:** 2014-06-10

**Authors:** Sarah E. Headland, Hefin R. Jones, Adelina S. V. D'Sa, Mauro Perretti, Lucy V. Norling

**Affiliations:** 1 The William Harvey Research Institute, Barts and The London School of Medicine, Queen Mary University of London, Charterhouse Square, London EC1M 6BQ, United Kingdom

## Abstract

Interest in extracellular vesicle biology has exploded in the past decade, since these microstructures seem endowed with multiple roles, from blood coagulation to inter-cellular communication in pathophysiology. In order for microparticle research to evolve as a preclinical and clinical tool, accurate quantification of microparticle levels is a fundamental requirement, but their size and the complexity of sample fluids present major technical challenges. Flow cytometry is commonly used, but suffers from low sensitivity and accuracy. Use of Amnis ImageStream^X^ Mk II imaging flow cytometer afforded accurate analysis of calibration beads ranging from 1 μm to 20 nm; and microparticles, which could be observed and quantified in whole blood, platelet-rich and platelet-free plasma and in leukocyte supernatants. Another advantage was the minimal sample preparation and volume required. Use of this high throughput analyzer allowed simultaneous phenotypic definition of the parent cells and offspring microparticles along with real time microparticle generation kinetics. With the current paucity of reliable techniques for the analysis of microparticles, we propose that the ImageStream^X^ could be used effectively to advance this scientific field.

The ability of a cell to respond to external stimuli by producing extracellular vesicles (an umbrella term including microparticles, exosomes and apoptotic bodies) is now appreciated^1^,^2^,^3^. These small structures, far from being inert cellular debris, play roles in physiological processes such as coagulation^4^ and contribute to the pathogenesis of a variety of diseases including atherosclerosis^5^,^6^, rheumatism^7^,^8^ and malignancy^9^. Furthermore, these microparticles may be harnessed as therapeutic vectors, increasing potency of soluble proteins^10^,^11^ and drugs^12^. However, knowledge on size, concentration, cell of origin, morphology and biochemical composition of extracellular vesicles, such as microparticles, is limited, due to their sub-micrometer size and the intrinsic limitations in methods applied for their characterization^1^.

Flow cytometry represents the mainstay of microparticle measurements in the literature, probably due to its ease-of-access. Recent developments in this field have progressed flow cytometry towards a standard for microparticle research^13^,^14^, yet the resolution of most commercially available cytometers remains around 500 nm. The methods adopted, such as calibration of the forward-scattering intensity using polystyrene microspheres is beset with issues regarding the refractive index relationship between size and material^15^. The use of high-resolution flow cytometry has enhanced the measurements obtainable using this method, and the Apogee A40 flow cytometer is able to resolve cell derived particles down to 400 nm in diameter^16^ using the forward scatter detector.

Advances in standard flow cytometric analysis are slow to be adopted. Whilst fairly robust for the measurement of cells, the forward scatter detector gives a variable signal between instruments, affecting reproducibility between laboratories. It is the most alignment-critical detector within the cytometer and can be affected by multiple variables, including mismatches in the refractive index between the sheath fluid and sample, beam geometry, polarization, stop position and collection angle. The forward scatter position of microparticles of different sizes does not therefore follow their relative order in physical size. Furthermore, resolving two microparticles requires a difference in size between them of at least 280 nm^17^. In contrast, side-scatter or “right-angled light scatter” shows good correlation to size. This is because all instruments aim for maximal light collection and are therefore designed to achieve similar numerical apertures. This makes the side-scatter a more robust discriminator, especially between instruments^17^. Side-scatter information teamed with fluorescence labeling allows further discrimination between cell fragments, noise and true microparticles. Setting the axis parameters to side-scatter versus fluorescence allows the more accurate placement of 0.5 μm beads and eliminates background noise from overlapping with the microparticle populations.

A second practical obstacle in the multi-labeling of microparticle samples is coincidence. The fluidics of flow cytometers is designed for passing single cells in a hydro-dynamically guided stream through the cuvette. Single isolated polymorphonuclear cells, as one example, occupy 630 μm^3^^18^, whereas microparticles are much smaller, resulting in sub-optimal hydrodynamic focusing. This allows multiple microparticles to be interrogated simultaneously and processed as a single event, especially in concentrated samples where double, triple, or more, false-positive single events may be recorded. Performing dilutions of the sample so that microparticles travel more rarely through the cuvette may aid in the reduction in coincidence, but conversely it would increase inter-sample variability further.

Other measurement assays are available for the analysis of microparticles, including optical methods such as nanoparticle tracking analysis, and non-optical methods like transmission electron microscopy, impedence-based measurements and Raman spectroscopy. Caveats to all of these techniques exist. Nanoparticle tracking analysis has a functional measurement cut-off at 1 μm, skewing sizing results towards small particles. Transmission electron microscopy, especially teamed with cryo-preparation has yielded superior results on the sizing, morphology and biochemical composition of microparticles^1^ but requires high-level skill and expertise for sample processing and analysis. It is not a technique that can be rapidly deployed in many laboratories and, even less, clinical settings.

We have used the ImageStream^X^ Mk II imaging cytometer (thereafter IS^X^ for short) for the detection of microparticles for the first time. The overwhelming advantage of this technology is the visual interrogation of each event passing through the flow cell. Even below the physical optical resolution cut-off of 200 nm, the IS^X^ can detect fluorescent signals of otherwise undetectable microparticle populations. We have evaluated presence of microparticles in a variety of blood-based samples, and provided phenotypic quantification of healthy plasma microparticles to demonstrate the superior usability of this technique. Herein, we describe for the first time the temporal kinetics of real-time microparticle generation from neutrophils and explored the role of the cytoskeleton in their production. We propose the use of this imaging cytometer will advance extracellular vesicle research, and has the potential to allow wide-scale adoption for the enumeration of microparticles from archived frozen plasma samples.

## Results

We have used the high sensitivity imaging cytometer, the Amnis ImageStream^X^ Mk II (IS^X^) for characterization of microparticle populations. This cutting edge instrument uses proprietary charge-coupled devices (CCDs) instead of photomultiplier tubes and captures up to 12 images (10-colour) of each event as it passes through the flow cell. IS^X^ was designed to allow the co-localization of subcellular particles within cells and is equipped with a X60 objective in order to do this, which subsequently makes it amenable to microparticle detection and analysis. Acquisition parameters can be determined by a huge variety of physical parameters rather than just size, complexity and fluorescence intensity. In terms of microparticle research, this analyzer represents important advancements in sample detection and measurement. Firstly, each event recorded is paired with a bank of images generated for each single event. Dot plots and image galleries are therefore searchable and can be used to corroborate event identity by eye. Multicolor staining can be further exploited for accurate identification of various subpopulations.

**Figure 1:**
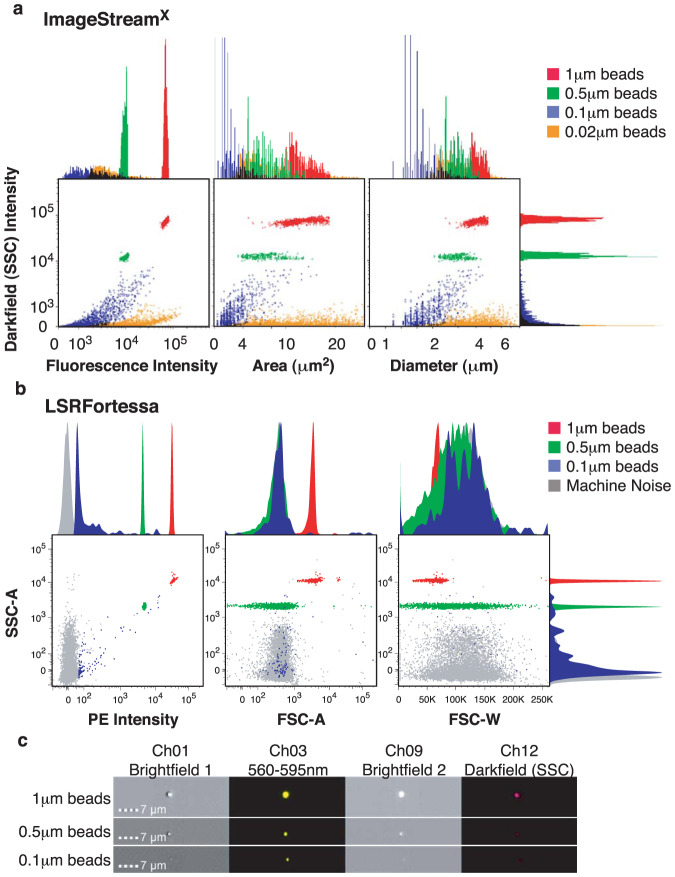
Sizing beads (1 μm, 0.5 μm, 0.1 μm and 0.02 μm) are differentiated using ImageStream^X^ Mk II imaging cytometer, which has superior detection compared to the BD LSRFortessa. Fluorescent latex beads of 1 μm, 0.5 μm, 0.1 μm and 0.02 μm were diluted in double-sterile filtered PBS and acquired using IS^X^ at X60 magnification. (a) The Dark Field Scattering intensity was plotted against fluorescence intensity (Channel 02 and Channel 11 for 0.02 μm beads; left hand panel), area (middle panel) or diameter (right hand panel), with measurements taken from the fluorescence area mask (70% saturation), with corresponding histograms shown. (b) The same bead samples were acquired using the BD LSRFortessa with low flow rate. Equivalent plots and histograms are shown. The presence of machine noise is indicated in grey, which is notably not present in the IS^X^. The smallest beads, 0.02 μm (orange), were completely undetectable using the BD LSRFortessa. (c) Representative images of beads of each size showing fluorescence intensity and brightfield images from brightfield camera 1 and 2, generated using the IS^X^. Although the 0.02 μm beads were detectable using the IS^X^, they could not be seen in the corresponding image banks.

Initially, we compared the sensitivity of the IS^X^ with a commercially available, standard flow cytometer, the BD LSRFortessa, using fluorescent 20 nm to 1 μm latex calibration beads. Importantly, we were able to detect 20 nm beads using the IS^X^ that were completely undetectable using the LSRFortessa. We found the best resolution of the two larger bead populations using the fluorescence intensity plotted against side-scatter for both machines (Figure 1a,b). We could also detect a larger number of 0.1 μm beads using the IS^X^, but more of this population was lost within the machine noise of the LSRFortessa. By contrast, no noise events were detected using the IS^X^. Sizing with IS^X^ according to area or diameter measurements were somewhat less useful, even when measured on the fluorescence channel with stringent masking. This is because, at such high magnification, a single pixel represents an area of 0.3 μm^2^, which is a large proportion of a 0.5 μm diameter bead or microparticle. Fluorescence signals also suffer from significant haloing, leading to an overestimation of particle size (Figure 1c). Nevertheless, the side-scatter measurements still corresponded to the relative size of the bead populations (Figure 1a, see histograms), and that the 20 nm beads were detectable by their fluorescent signal suggests that the enumeration of microparticles of all sizes is feasible. In contrast, the LSRFortessa struggled with forward-scatter laser measurements, and the beads could not be placed according to size, and the smallest beads were undetectable.

Furthermore, the machine's advanced fluidics control and reliability of the Objects per ml feature could be demonstrated by serial dilution of microparticle samples. Indeed, Pearson correlation coefficients close to 1 were achieved for three donors between sample dilution and microparticles per ml (see Supplementary Fig. S1 online). Testament to the fine fluidics control of the IS^X^, coincidence, or “swarms” of microparticles or beads were also not encountered at any dilutions of the sample acquisition.

**Figure 2:**
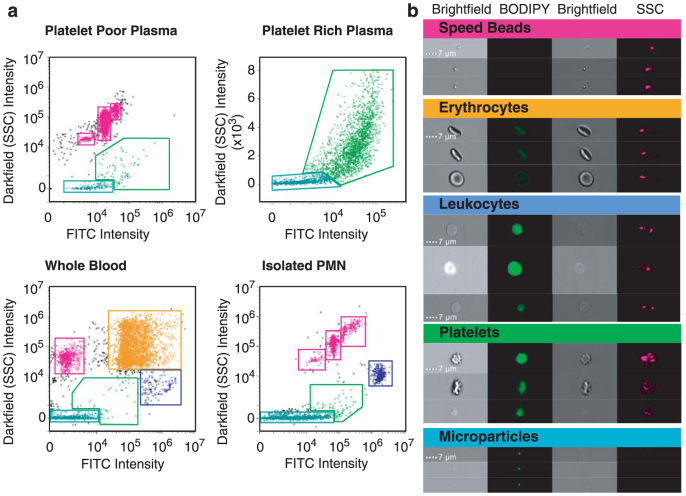
Distinct populations of cells, platelets and microparticles can be differentiated within whole blood, plasma and isolated cells. (a) Platelet-poor and platelet-rich plasma, whole blood and isolated TNF-α-stimulated polymorphonuclear (PMN) cell samples were labeled with BODIPY-Maleimide dye prior to acquisition on the IS^X^. Pink gates denote speed beads (and their aggregates), used as internal calibration for the cytometer; orange gate denotes erythrocytes; blue gate denotes leukocytes (neutrophils); green gate denotes platelets and aqua gate denotes microparticles, that appear as low scatter, low fluorescence intensity. (b) Representative images from each of the cell population gates.

Next, we tested IS^X^ for microparticle detection in complex biological fluids such as platelet-poor and platelet-rich plasma, whole blood, and the supernatants of stimulated isolated neutrophils (Figure 2). Uniquely among analyzers used for detecting microparticles, the parent cells do not need to be removed before acquisition. By staining with BODIPY-maleimide dye, we could identify leukocyte, platelet, erythrocyte and microparticle populations without the need for phenotypic markers. Microparticles could be detected in all samples, at different quantities, and differed from the internal calibration beads (of similar size and BODIPY-Maleimide fluorescence intensity) due to their high side-scatter signal. BODIPY-Maleimide staining was adopted for these experiments as labeling is almost instant, and the cells do not require washing before sample acquisition. As a result, these speed beads also take up some of the dye and fluoresce, but still form discrete populations that can be gated out of the analysis. Advantageously, each event gated could be visually corroborated by interrogation of the images generated for each event (Figure 2b) thus, the large majority of events could be placed in one of each of the gates.

**Figure 3:**
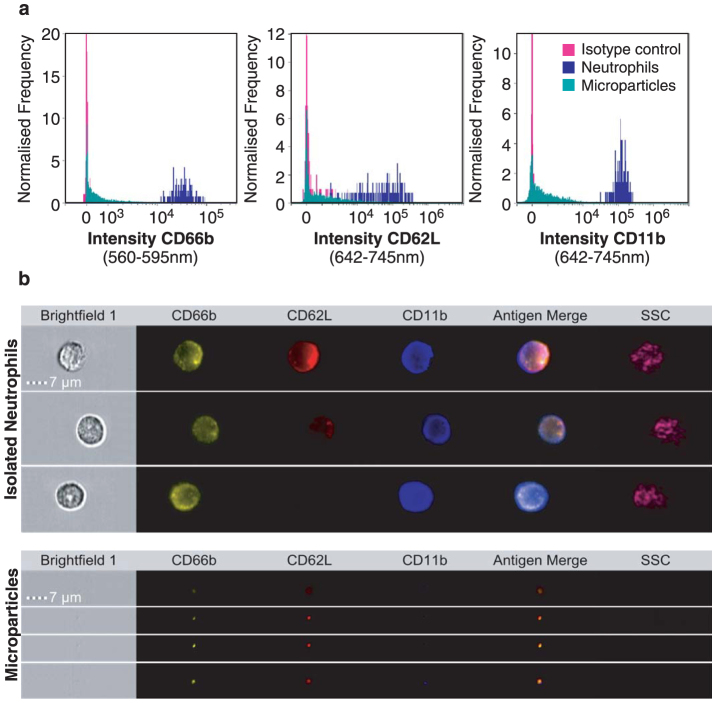
Simultaneous analysis of parent cell and microparticle phenotype. Isolated neutrophils were stimulated with TNF-α (50 ng/ml) for 20 minutes then stained on ice with anti-CD66b, anti-CD62L and anti-CD11b before acquisition using IS^X^. (a) Histograms of CD66b, CD62L and CD11b intensity of offspring microparticles and neutrophils, compared to isotype control (pink). (b) Representative images of labeled neutrophils (upper panel) showing increasingly activated phenotypes, and offspring microparticles (lower panel) with examples of double and triple-positive events.

As our analyses had relied only on pan-labeling of cells and microparticles by BODIPY-Maleimide, next we investigated whether we could simultaneously interrogate the activation status of TNF-α stimulated neutrophils using anti-CD66b, CD62L and CD11b staining, and whether these antigens could be detected on the offspring microparticles (Figure 3). As expected, neutrophils were highly positive for CD66b and with TNF-α stimulation shed a proportion of their L-selectin (CD62L), whereas CD11b was highly expressed (Figure 3a). Furthermore, the microparticles also displayed a high degree of CD66b positivity, and their CD62L expression overlapped with those of neutrophils and CD11b was also expressed. Figure 3b displays representative images; the uppermost neutrophil panel shows a neutrophil with intermediate CD66b, CD62L and CD11b expression, suggesting incomplete activation, the neutrophil in the lowest panel displays higher CD66b and CD11b expression with a complete loss of CD62L suggesting full activation.

**Figure 4:**
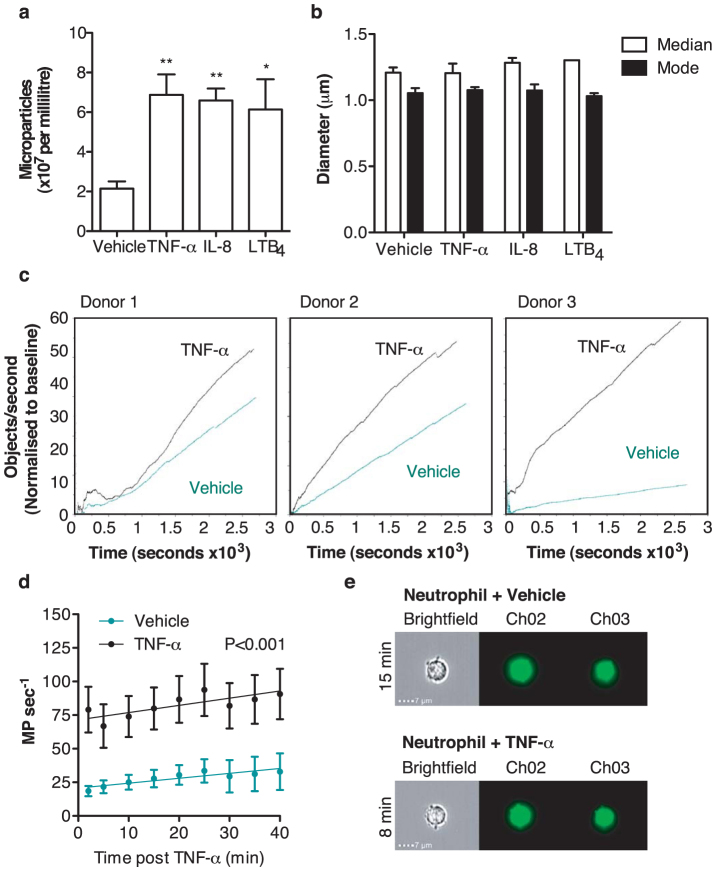
Isolated microparticles can be quantified and the real-time generation of neutrophil microparticles can be assessed with IS^X^. (a) Neutrophils were labeled with BODIPY-Maleimide, then microparticles were generated using TNF-α (50 ng/ml), IL-8 (50 ng/ml) or leukotriene B_4_ (10 nM) and enumerated using IS^X^. (b) Microparticle diameter was assessed using the Channel 02 fluorescence parameter with a 75% stringent mask applied. Data are shown as mean ± SEM of 3 blood donors. *P<0.05 and **P<0.01 using repeated measures ANOVA and Bonferroni post-test. (c) Neutrophils were isolated from three healthy donors, immediately labeled with BODIPY-Maleimide dye and stimulated with TNF-α (50 ng/ml) followed by immediate acquisition for 45 minutes. The rate of microparticle production was inferred by gating the microparticle population, applying the “Objects per second” feature and plotting against time. (d) Summarized data from the three donors, *P*<0.001 with Mixed-model ANOVA. (e) Representative IS^X^ gallery images of neutrophil microparticles budding at the times indicated.

As Timar, et al.^19^ had demonstrated that differential stimulation of neutrophils leads to different numbers of microparticles being generated, we used the IS^X^ to enumerate the number of microparticles produced during cell activation (Figure 4a). This analyzer has syringe driven fluidics and achieves a high level of control over the flow rate passing through the flow cell using the speed beads for calibration. Therefore it can achieve accurate concentration measurements. Following 20-min stimulation of neutrophils with TNF-α (50 ng/ml), IL-8 (50 ng/ml) and leukotriene B_4_ (LTB_4_; 10 nM), microparticle production doubled compared with resting cells (TNF-α 6.9 ± 1 *vs*. vehicle 2.1 ± 0.4 × 10^7^ microparticles/ml from 2.5 × 10^6^ neutrophils in 50 μl, N = 3; *P*<0.01 with Repeated measures ANOVA and Bonferroni post-test; LTB_4_ 6.1 ± 1.5 and IL-8 6.6 ± 0.6 *vs.* vehicle 2.1 ± 0.4 × 10^7^ microparticles/ml from 2.5 × 10^6^ neutrophils in 50 μl, n = 3; *P*<0.05 with Repeated measures ANOVA and Bonferroni post-test). The approximate diameter of the offspring microparticles did not alter upon stimulation (Figure 4b).

To explore microparticle generation further, IS^X^ analysis was employed to quantify neutrophil microparticle production in real time. As IS^X^ analysis allows parallel analysis of both parent neutrophils and offspring microparticles in the same sample collection, it proved a powerful tool for assessing the kinetics of microparticle formation. IS^X^ analysis of neutrophils labeled with BODIPY-Maleimide and stimulated with TNF-α (50 ng/ml) immediately prior to acquisition revealed that the rate of neutrophil microparticle production was significantly higher when the cells were stimulated with TNF-α (50 ng/ml) than that of resting cells over a 45-minute period (Figure 4c,d; *P*<0.001 with Mixed-model ANOVA). It was possible to observe budding neutrophil microparticles during this analysis (Figure 4e) and therefore IS^X^ analysis of both parent and offspring microparticles, concurrently within one sample, lends itself to multiple applications.

**Figure 5:**
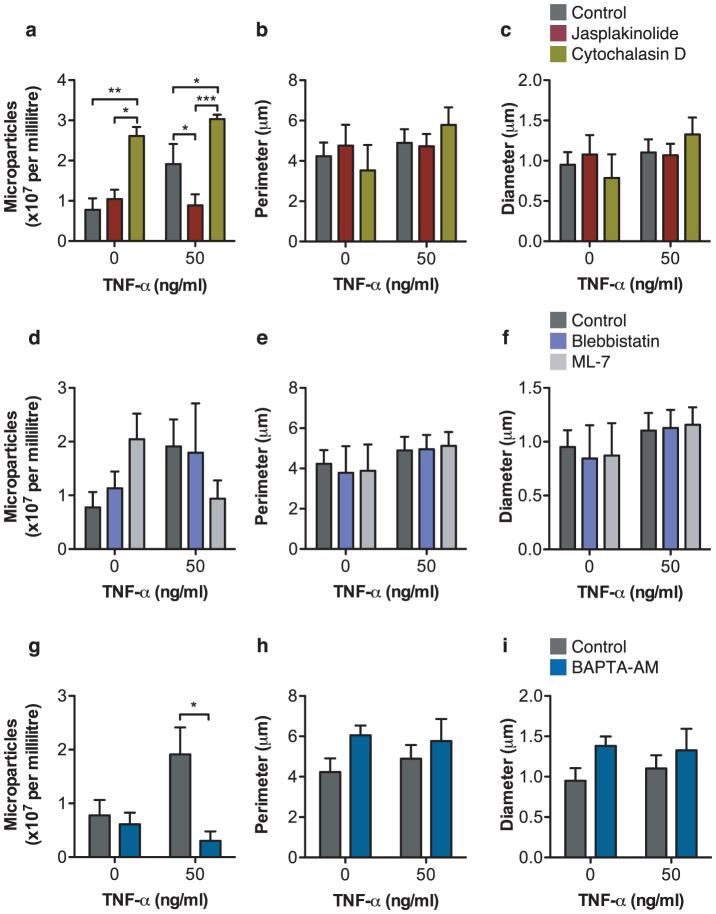
The mode of microparticle shedding is largely actin-dependent and requires intracellular calcium. Neutrophils isolated from 6 healthy donors were treated with drugs that interfere with actin polymerisation (Jasplakinolide, 1 μM and Cytochalasin D, 2 μM; panels a–c) or myosin-dependent cell contractility (Blebbistatin, 10 μM and ML-7, 20 μM; panels d–f) and a calcium chelator (BAPTA-AM, 20 μM; panels g–i) before the addition of TNF-α (50 ng/ml) for 20 minutes. Cells were removed by gentle centrifugation, and microparticles were stained with BODIPY-Maleimide dye before acquisition using IS^X^. Offspring microparticles were enumerated (panels a, d and g) and the perimeter (panels b, e and h) and diameter (panels c,f and i) were measured following the application of a fluorescence intensity mask applied to Channel 02 (BODIPY-Maleimide fluorescence) following erosion to 70% to ensure tight masking. *P<0.05, **P<0.01 and ***P<0.001 using Matched Two-way ANOVA with Bonferroni post-test.

The cytoskeletal control of microparticle production was next assessed. Microparticle generation requires cytoskeletal re-organization in the origin cell. We applied distinct compounds to dissect the involvement of specific intracellular events in microparticle formation, using Jasplakinolide and cytochalasin D to stabilize and destabilize the actin cytoskeleton, respectively (Figure 5a–c). We targeted the interaction of actin and myosin using blebbistatin (non-muscle myosin II inhibitor preventing cell contraction allowing blebbing) and ML-7 (a selective myosin light-chain kinase inhibitor; Figure 5d–f) and finally chelated the intracellular calcium of neutrophils with BAPTA-AM before stimulation with TNF-α (50 ng/ml; Figure 5g–i) to induce microparticle shedding. As neutrophil viability was not significantly affected by the drugs tested (data not shown), the offspring microparticles were isolated by sequential centrifugation to deplete the cells, loaded with BODIPY-Maleimide dye and quantified using ImageStream^X^ analysis. Jasplakinolide, which inhibits actin depolymerization and cytochalasin D, which inhibits actin polymerization, both had significant effects on the number of microparticles produced by neutrophils without altering the size or shape of the microparticles generated. Jasplakinolide significantly inhibited microparticle production induced by TNF-α stimulation: 0.9 ± 0.3 *vs.* 1.9 ± 0.5 × 10^7^ microparticles/ml (n = 4; *P*<0.05 with Two-way ANOVA and Bonferroni post-test) whereas cytochalasin D increased microparticle production in unstimulated (2.6 ± 0.2 *vs.* 0.8 ± 0.3 × 10^7^ microparticles/ml, *P*<0.01 with Two-way ANOVA and Bonferroni post-test) as well as TNF-α stimulated neutrophils (3.0 ± 0.1 *vs.* 1.9 ± 0.5 × 10^7^ microparticles/ml, *P*<0.05 with Two-way ANOVA and Bonferroni post-test), and induced the highest amount of microparticle shedding of all the drugs tested. These data confirm the role of the actin cytoskeleton in the regulation of microparticle production, as enhanced actin polymerization inhibits microparticle shedding and disruption of the cortical actin network enhances the number of microparticles generated. In contrast, treatment with drugs that inhibit non-muscle myosin motors did not significantly modify microparticle numbers from seven individual blood donors (Figure 5d). The size of the microparticles was also not altered (Figure 5e,f). This suggests that microparticle formation can occur in the absence of myosin-induced cell contraction.

Intracellular fluxes of calcium are known to be heavily involved in the events surrounding microparticle formation and shedding from initial receptor ligation up to the rearrangement of cytoskeletal components and phospholipid reorganization. Calcium fluxes also recruit annexins to the plasma membrane to achieve membrane cleavage and microparticle release^2^. Intracellular calcium chelation using BAPTA-AM significantly inhibited microparticle production compared to stimulation with TNF-α (0.3 ± 0.2 *vs.* 1.9 ± 0.5 × 10^7^ microparticles/ml, N = 6; *P*<0.05 with Two-way ANOVA and Bonferroni post-test) as expected (Figure 5g).

**Figure 6:**
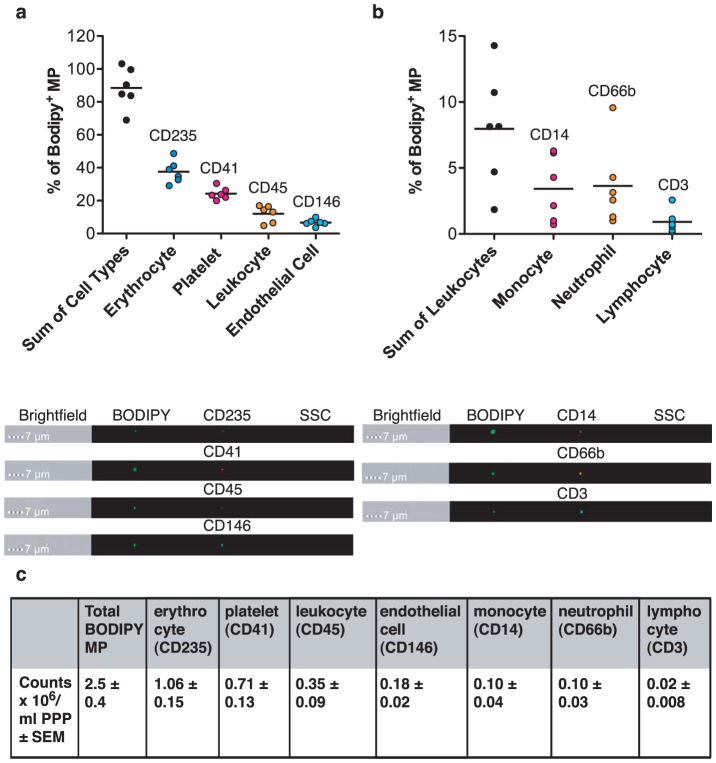
Plasma microparticles from healthy human donors are largely derived from erythrocytes and platelets. (a) Platelet-poor plasma was generated from 6 healthy donors and stained to determine the microparticle cell of origin using CD235 (erythrocyte), CD41 (platelet), CD45 (leukocyte) and CD146 (endothelial cells) markers. (b) Leukocyte microparticles were further phenotyped to determine their cell of origin using CD14 (monocyte), CD66b (neutrophil) and CD3 (lymphocyte) markers. Data are expressed as percentage BODIPY-Maleimide positive events and shows mean (line) and individual raw values. Below are representative images of events from the gallery. (c) Table shows absolute counts ± SEM of N = 6 donors.

Finally, we quantified the number of circulating microparticles in healthy donor samples and elucidated their cell of origin (Figure 6a and b). The attractive possibility of using circulating microparticle levels as putative biomarkers for disease states requires the thorough testing of healthy donors to define “normal” limits, and agreement on the number of circulating microparticles is lacking^13^,^20^, probably due to the variety in techniques applied. We generated platelet-poor plasma samples from 6 healthy donors and interrogated their cell of origin using conjugated antibodies to antigens previously reported to be shed onto microparticles^21^,^22^,^23^,^24^,^25^,^26^,^27^. The entire microparticle population was labeled by the addition of BODIPY-Maleimide dye and double-positive events expressed as a percentage of the BODIPY-Maleimide labeled population (Figure 6a and b). Thus, in six distinct preparations the majority of circulating microparticles were observed to be of erythrocyte origin (37.5%), then of platelet (24.3%), leukocyte (12%), and endothelial cell origin (6.6%). We subtyped the leukocyte population further and found that neutrophil microparticles comprised the largest proportion of leukocyte microparticles (3.6%) then monocyte (3.4%) and lymphocyte microparticles (0.9%). The sum of the leukocyte-positive microparticle proportions equaled 8%; lower than the CD45^+^ leukocyte microparticle population at 12%. This discrepancy could represent a proportion of leukocyte microparticles of an unaccounted origin, or that not all antigen transfer onto microparticles is equal. In terms of microparticle counts, we found that the average total microparticle count per milliliter of platelet poor plasma was 2.5 × 10^6^ (Figure 6c).

## Discussion

At present, the high-throughput, high-accuracy enumeration and measurement of microparticles requires multiple techniques for production of robust and reproducible data. For example, commercial flow cytometers have a lower-limit of detection that excludes many of the smaller particles from sample analysis, and is of low accuracy^16^. Nanoparticle tracking analysis, which measures the Brownian motion of particles in suspension providing accurate quantification of microparticle number and size, has an upper detection-limit cut-off at 1 μm. The availability of this system with multiple color labeling is also not currently available. Specialist techniques such as atomic force microscopy, impedance-sensing and Raman spectroscopy also have caveats such as the relative expense and do not lend themselves to wider-reaching applications. The gold standard of cryo-transmission electron microscopy provides the most accurate data, but requires expert implementation^1^. We propose here that the ImageStream^X^ imaging cytometer can give accurate enumeration, allowing simultaneous detection of larger (≥1 μm) and smaller (approximately 20 nm) microparticle populations and parent cells. With this technology, kinetics of microparticle production could be accurately measured for the first time.

The IS^X^ was superior at detecting smaller sized latex beads compared to the LSRFortessa, and analysis using the IS^X^ benefits from the ability to visually interrogate each individual event passing through the flow cell, allowing corroboration of events presented in dot plots and histograms. Noise within the analyzer was also minimal compared to the LSRFortessa, due to both the multiple-interrogation of each event, and the self-sterilization and cleaning system within the setup. Furthermore, sample preparation, depending on the application required, can range from minimal, i.e. simple fluorescent dye labeling, to extensive, with panels of multiple antibodies. Here, we demonstrated the detection of microparticles in whole blood using BODIPY-Maleimide labeling, which could also be applied to plasma and isolated cells. We then demonstrated the applicability of phenotyping parent neutrophils and their offspring microparticles simultaneously. None of these experimental protocols have been possible before.

Microparticles bear antigens from their parent cells, allowing phenotypic measurements to be made about their parent cell of origin^28^. Not only did neutrophil microparticles express the canonical CD66b neutrophil marker used for microparticle phenotyping in previous studies, but they also bore CD62L and CD11b. In these analyses we could quantify simultaneously the activation status of the parent cells and their offspring microparticles. It was also possible to identify the proportions of microparticle subpopulations from healthy plasma. As microparticle analysis has a potential clinical use as a biomarker for a variety of diseases, normal limits need to be defined, thus detection of microparticles in platelet-poor plasma opens up the opportunity for use of frozen archived samples for microparticle analysis. We demonstrate here that quantification of microparticles (with or without defining their cell of origin) is possible with minimal sample preparation.

The real-time temporal kinetics of microparticle production has not before been studied in neutrophils. Static measurements of microparticle number have been made using aliquots of cerebral vascular endothelial cell line supernatant taken and tested by flow cytometry^29^ or by spectrophotometric analysis of the supernatants of adherent glial cells stimulated with an ATP mimetic^30^. During glial cell culture, stimulation with ATP induced a plateau of microparticle production after 20 minutes, and with time-lapse microscopy, microparticle shedding could be observed (but not quantified) 1–2 minutes after stimulation^30^. Reported here, analysis of freshly isolated neutrophils in suspension, stimulated with TNF-α and analyzed in real-time over 45 minutes showed an increase in the rate of microparticle production compared to resting cells. Although a plateau in microparticle production was not reached over this time-course, the limiting factor of data acquistion was the file size generated during such an experiment. This assay could be translated to explore the real-time kinetics of microparticle generation in combination with cytoskeletal inhibitors, such as those used in single time-point experiments.

The process of microparticle production and release (vesiculation) requires the dynamic coordination of cellular components for effective destabilization of the plasma membrane. It has been observed that during microparticle formation in cerebral microvascular endothelial cells, there is dissolution of the cortical F-actin network, followed by prominent actin stress fiber formation, and redistribution of vinculin to the newly formed fibers^29^. Consistent with these findings, our data show that treatment of neutrophils with jasplakinolide, which promotes actin polymerization and stabilizes actin filaments into an amorphous mass, inhibits microparticle release. In contrast, treatment with cytochalasin D, a potent inhibitor of actin polymerization, increasing the cellular membrane fluidity, increases microparticle release. As demonstrated with the IS^X^, this increase in microparticle shedding was not accompanied by concomitant increases in microparticle size. Disrupting the functions of non-muscle myosin to disrupt cell contraction with ML-7 and blebbistatin did not lead to alterations in the number of microparticles shed. In addition, microparticle release, along with many cellular processes, is calcium-dependent^31^. Intracellular calcium chelation led to blockade of microparticle shedding. This suggests that regulated microparticle formation is reliant on the ability of actin to polymerize and depolymerize in a coordinated fashion. Further real-time studies on the production of microparticles under conditions of cytoskeletal rearrangement inhibition will uncover further regulation.

We have enumerated microparticles from the plasma of 6 healthy human volunteers, but further work to expand the cohorts of normal subjects to define the “normal” limits of microparticle phenotypes is required. The ultimate goal of microparticle analysis for use in detecting pathology in clinical settings would be the direct enumeration of both leukocyte subsets and the microparticles they produce^32^. We provide evidence for the first time that this is achievable using the IS^X^. We were able to phenotypically identify the majority (>90%) of microparticles within the plasma samples; full identification of the remainder of “unknown” microparticles is, however, likely to remain asymptotic. As any cell type is capable of producing microparticles (the machinery required for microparticle release is likely to be similar to endosomal and vesicular transport), the characterization of the origin of microparticles will require extensive exploration in the biology of this field, even with advances in technology. Clearly, this analyzer has many potential diagnostic applications, but this novel combination of cellular and microparticle enumeration makes the IS^X^ a powerful tool, for the future of microparticle research.

## Methods

### Materials

ImageStream^X^ Mk II was from Amnis Corporation, Seattle, WA, USA and the BD LSRFortessa from BD Biosciences (San Jose, California). All chemicals and reagents were purchased from Sigma-Aldrich, Poole, UK, unless otherwise stated, including 0.1 μm (L1528-1ML), 0.5 μm (L1403-1ML) and 1 μm (L1278-1ML) latex calibration beads, and the cytoskeletal inhibitors. Crimson fluorescent 0.02 μm FluoSpheres (F-8782), Dextran (MW 450,000–650,000), cytochalasin D, histopaque 1077 and BODIPY FL N-(2-aminoethyl)maleimide) (FITC and Texas Red BODIPY-Maleimide) were purchased from Life Technologies, Invitrogen, Paisley, UK. Leukotriene B_4_ was purchased from Cayman Chemical, Ann Arbor, MI, USA. Antibodies were used as follows: anti-human CD66b-PE (BioLegend, clone G10F5) at 0.25 μg/ml with isotype IgM (BioLegend, clone MM-30); anti-human CD11b-pacific blue (BioLegend, clone ICRF44) used at 10 μg/ml with isotype IgG1 (BioLegend, clone MOPC-21); anti-human CD62L-PE/Cy5 (BioLegend, clone DREG-56) used at 1 μg/ml with isotype IgG1 (BioLegend, clone MOPC-21); anti-human CD235a-FITC (eBioscience, clone HIR2) used at 0.25 μg/ml with isotype IgG2b (eBioscience, clone eBMG2b); anti-human CD146-APC (BioLegend, clone SHM-57) used at 0.5 μg/ml with isotype IgG2a (BioLegend, clone MOPC-173); anti-human CD45-PerCP/Cy5.5 (eBioscience, clone HI30) used at 0.5 μg/ml with isotype IgG1 (eBioscience, clone P3.6.2.8.1); anti-human CD41-FITC (BioLegend, clone HIP8) used at 0.25 μg/ml with isotype IgG1 (BioLegend, clone MOPC-21); anti-human CD14-PE/Cy7 (BioLegend, clone HCD14) used at 2 μg/ml with isotype IgG1 (BioLegend, clone MOPC-21); anti-human CD66b-FITC (AbD Serotec, clone 80H3) used at 1/100 dilution with isotype IgG1 (BioLegend, clone MOPC-21); anti-human CD3-APC (BioLegend, clone UCHT1) used at 0.5 μg/ml with isotype IgG1 (BioLegend, clone MOPC21).

### Methods

#### Preparation of calibration beads

Latex beads were diluted 1:15,000 in double-sterile-filtered (0.22 μm) PBS after 5-minute sonication and vortexing to disperse. Samples were acquired immediately with either the BD LSRFortessa at low flow rate, or using IS^X^ as described below.

#### Preparation of samples

All volunteers gave written informed consent to blood collection and the procedure was approved by the East London & The City Local Research Ethics Committee (Rec Ref. 05/Q0603/34 ELCHA, London, United Kingdom) in accordance with the World Health Organization guidelines on drawing blood. Peripheral blood from healthy donors was collected by intravenous withdrawal and added to sodium citrate solution to prevent coagulation. Neutrophils were isolated from blood via density centrifugation on a Histopaque 1077 gradient (Sigma Aldrich) according to the manufacturer's instructions. In detail, blood (60 ml) was taken from healthy volunteers using a 21G butterfly needle with tourniquet applied and anticoagulated with 0.32% w/v sodium citrate. Whole blood was stained as below. Platelet-rich and platelet-poor plasma were generated by centrifugation at 300 × *g* for 20 minutes to pellet the cells, then the platelet rich plasma recovered, and platelets depleted by a third centrifugation at 10,000 × *g* for 2 minutes. Neutrophils were isolated from whole blood using dextran sedimentation followed by density gradient separation as follows: blood was centrifuged at 130 × *g* for 20 minutes at room temperature to sediment the cells. Platelet rich plasma was removed before 10 ml phosphate-buffered saline (PBS) was carefully layered over the cell suspension, to avoid mixing. Dextran (from *Leuconostoc*, molecular weight 450,000 to 650,000; 6% w/v, 8 ml) was gently layered onto the PBS. The tubes were inverted gently to mix the cell suspension with the PBS and dextran. After 20 minutes the leukocyte rich layer was carefully collected, and layered over 10 ml Histopaque 1077 in a fresh 50 ml falcon tube and centrifuged for 30 minutes at room temperature at 450 × *g*.

The supernatant was aspirated and the red blood cells were lysed through hypotonic shock with 9 ml ice-cold ultrapure distilled water. Isotonicity was quickly restored by adding 1 ml 10x Hanks Balanced Salt solution. Granulocytes were pelleted at 300 × *g* for 10 minutes at room temperature, the supernatant was aspirated and the granulocyte pellet was resuspended in phenol red-free RPMI and adjusted to 2 × 10^7^/ml for stimulation.

For fixed-time microparticle generation, cells (2 × 10^7^/ml) were stimulated with TNF-α (50 ng/ml, Sigma), IL-8 (50 ng/ml, Peprotech), LTB_4_ (10 nM, Cayman Chemical) or vehicle (PBS) for 20 minutes at 37°C before placing on ice to arrest microparticle production. Cell suspensions were then either processed for ImageStream^X^ analysis or their neutrophils were pelleted by centrifugation at 4500 × *g* for 15 minutes at 4°C followed by a second centrifugation at 13,200 × *g* at 4°C for 2 minutes. Samples were resuspended in phenol-red free RPMI before proceeding to processing for ImageStream^X^ analysis.

For real-time microparticle generation, freshly isolated neutrophils were resuspended at 1 × 10^6^ cells/ml in phenol red-free RPMI and labelled with BODIPY-Maleimide dye (2.5 μM) 2 minutes prior to sample acquisition, then the sample loader lowered. TNF-α (50 ng/ml) was added immediately prior to placing the eppendorf into the sample loader and loading. Acquisition of events was started immediately, and the events appearing in the microparticle population gate collected on low speed/high sensitivity for 45 minutes.

In other experiments, the phenotype of neutrophils was assessed. Isolated neutrophils were stimulated with TNF-α (50 ng/ml) for 20 min at 37°C then stained with CD66b-PE, CD62L-PE/Cy5 and CD11b-pacific blue for 30 min on ice. Samples were then acquired with IS^X^.

For circulating plasma microparticle phenotyping experiments, 5 μl platelet poor plasma PPP was diluted 1:2 with human immunoglobulin gamma (8 mg/ml final; Sigma) to block Fc receptors for 10 minutes. Samples were then stained with the following antibodies: 50 μM BODIPY-Maleimide – Texas Red, CD235-FITC (erythrocyte marker), CD45-PerCP/Cy5.5 (leukocyte marker), CD146-APC (endothelial marker), CD41-FITC (platelet marker), CD14-PE/Cy7 (monocyte marker), CD66b-FITC (neutrophil marker), CD3-APC (lymphocyte marker). Appropriate isotype and single stain controls were used. Samples were stained in the dark at room temperature for 30 minutes before acquisition with IS^X^.

#### Effect of cytoskeletal inhibitors

To investigate the intracellular machinery required for microparticle generation, freshly isolated neutrophils were treated for 10 minutes with Cytochalasin D (2 μM), Jasplakinolide (1 μM), Blebbistatin (10 μM), ML-7 (20 μM) or 1,2-*Bis*(2-aminophenoxy)ethane-*N,N,N',N'*-tetraacetic acid tetrakis(acetoxymethylester) (BAPTA-AM; 20 μM) at 37°C. Neutrophils were then plated into 96-well plates and centrifuged at 200 × *g* for 30 seconds at room temperature, followed by washing in PBS. Cells were then stimulated with TNF-α (50 ng/ml) or vehicle (PBS) for 20 minutes. Supernatants were collected by centrifugation at 3500 × *g* to deplete neutrophils, stained with BODIPY-Maleimide dye as above and acquired with the ImageStream^X^ Mk. II to quantify microparticle number and size.

#### Analyses on the ImageStream^X^ MkII

All samples were acquired on an ImageStream^X^ MkII imaging cytometer, X60 magnification; with low flow rate/high sensitivity using INSPIRE software. In order to detect microparticles in the samples, and to visualize the speed beads running throughout acquisition, the setting “Hide Beads” under the Advanced>Acquisition menu had to be unchecked. The percentage of BODIPY-Maleimide^+^ microparticles positive for each lineage marker was measured.

The instrument and INSPIRE software were set up as follows: Channels 01 (bright field), 06 (bright field 2) and channel 12 (scattering channel), plus fluorescence channels required. Magnification was 60X, providing a pixel size of 0.3 μm^2^ and the lasers 488 and 745 activated for fluorescence and side-scatter, respectively; with or without 405 and 642 laser activation. The flow rate was set to low speed/high sensitivity and stream alignment was adjusted where necessary. To obtain a collection gate, a scatter plot of BODIPY-Maleimide (channel 02 for FITC-conjugated, channel 04 for Texas Red conjugated) fluorescence intensity was plotted against side scatter (channel 12) side scatter intensity. This allows discrimination of speed beads, which are used as an internal calibrator for the machine's image capture system to determine the flow rate. These speed beads run continuously throughout both sample collection and while the machine is idle, but could be completely excluded from microparticle analysis as they form a discrete high-scatter intensity, low channel 02 fluorescence intensity population.

BODIPY-Maleimide labeled microparticles appeared as a low-scatter, low to mid fluorescence intensity, and any contaminating cells appear as high fluorescence intensity. Each gated population was interrogated via the image gallery to determine the upper and lower limits of microparticle size and shape. Microparticles characteristically appear as small, spherical FITC or Texas Red-fluorescent points, and can be discriminated from cells, which are much larger, or cellular debris, which is not uniformly spherical.

For real-time neutrophil microparticle generation experiments, the upper collection event gate was set to 1 × 10^6^ to prevent premature termination of sample acquisition. For simple enumeration of pre-prepared microparticle samples, the acquisition cut-off was set to 10,000. To determine the concentration of the sample, IDEAS software was used. Raw image files with the entire number of events were opened. A scatter plot showing channel 02 fluorescence intensity plotted against scattering intensity of channel 12 was generated and a microparticle gate re-applied and inspected visually to exclude inappropriate events. The gate was adjusted where necessary. The objects/mL feature was added to the analysis area, and applied to the microparticle gate under channel 02 fluorescence, after the default area mask had been adjusted to around 75%. This was more sensitive at capturing microparticles than the bright field as significant haloing contributes to the sample fluorescence, augmenting the signal. Other parameters, such as diameter and perimeter were determined by addition to the features list, after adjusting the mask in channel 02 to fit tightly around the event. To analyze the number of microparticles generated over the 45-minute sample acquisition, all events falling in the microparticle gate were plotted on a scatter graph showing time versus objects/sec. Each plot was manually adjusted so that the machine noise generated at the beginning of acquisition was set to zero, allowing determination of the rate of microparticle production over time, regardless of the number of microparticles that were already present within the sample.

#### Statistical analysis

Statistical analysis was carried out using SPSS 22 (IBM). Mixed-model ANOVA was used for microparticle generation, One-way repeated measures ANOVA with Bonferroni post-test was applied to number of microparticles generated from neutrophils during stimulation with stimuli. Two-way repeated measures ANOVA with Bonferroni post-test was applied during cytoskeletal inhibition experiments. A *P* value of <0.05 was considered significant.

## Author Contributions

S.E.H. designed experiments, collected and analyzed data and wrote the manuscript. H.R.J. designed experiments, collected and analyzed data and contributed to the manuscript. A.S.V.D. collected data. M.P. and L.V.N. contributed to the manuscript, coordinated experiments and oversaw the project. All authors reviewed the manuscript.

## Supplementary Material

Supplementary Information Supplementary Info
