# Supplementary material for: Cutting-Edge Analysis of Extracellular Microparticles using ImageStreamX Imaging Flow Cytometry
Source: Sci Rep. 2014 Jun 10;4:5237. doi: 10.1038/srep05237 (PMC4050385; doi:10.1038/srep05237)
Supplement: Supplementary Information — Supplementary Info [file srep05237-s1.pdf]

Supplemental File

Cutting-Edge Analysis of Extracellular Microparticles using ImageStream^X Imaging Flow Cytometry

Sarah E Headland[1], Hefin R Jones[1], Adelina SV D'Sa[1], Mauro Perretti[1], Lucy V Norling[1]

[1]The William Harvey Research Institute, Barts and The London School of Medicine, Queen Mary University of London, Charterhouse Square, London EC1M 6BQ, United Kingdom.

Author for correspondence: Mauro Perretti, Centre for Biochemical Pharmacology, The William Harvey Research Institute, Barts and The London School of Medicine and Dentistry, Queen Mary University of London, Charterhouse Square, London EC1M 6BQ, United Kingdom.

Email:

m.perretti@qmul.ac.uk;

Phone no: +44-2078828783; Fax no: +44-207-8826076

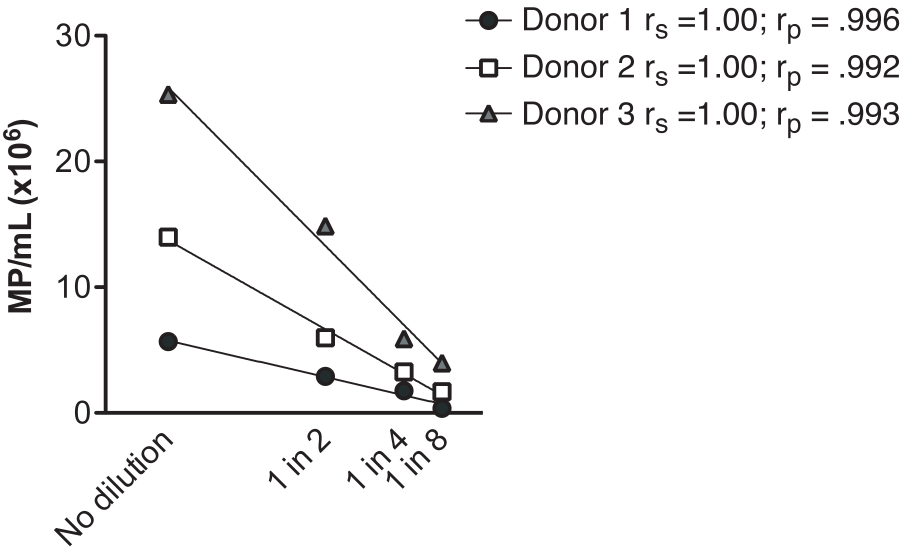

Dilution	Microparticles/ml (x10^6)		
	Donor 1	Donor 2	Donor 3
1	5.68	13.99	25.34
1:2	2.91	5.98	14.88
1:4	1.77	3.26	5.90
1:8	0.37	1.68	3.97

Supplementary Figure S1. The correlation of microparticle number versus sample dilution using the ImageStream^X is 1. Serially diluted microparticle samples (in double-sterile 0.22μm-filtered PBS) from 3 separate donors were stained with BODIPY-Maleimide before acquisition using the IS^X. Microparticle numbers were calculated using the objects/ml feature in IDEAS software (shown in table) and were plotted against dilution. r_s is Spearman's rho; r_p is Pearson's correlation coefficient.
